# Distribution of class 1 integrons in historic and contemporary collections of human pathogenic *Escherichia coli*

**DOI:** 10.1371/journal.pone.0233315

**Published:** 2020-06-02

**Authors:** Susanne Sütterlin, James E. Bray, Martin C. J. Maiden, Eva Tano

**Affiliations:** 1 Department of Zoology, University of Oxford, Oxford, United Kingdom; 2 Department of Women’s and Child’s Health, International Maternal and Child Health (IMCH), Uppsala University, Uppsala, Sweden; 3 Department of Medical Sciences, Uppsala University, Uppsala, Sweden; Cornell University, UNITED STATES

## Abstract

Integrons play a major role in the evolution and spread of antimicrobial resistance in human pathogens, including *Escherichia coli*. This study describes the occurrence of class 1 integrons in human pathogenic *E*. *coli*, in three isolate collections involving three periods from the last 100 years (i) the Murray collection (n = 58 bacteria isolated from the 1910s to 1940s); (ii) the *E*. *coli* reference (ECOR) collection (n = 37 isolates mainly from the 1980s); and (iii) a recently assembled collection (n = 88 isolates obtained in 2016). High-quality whole genome sequences (WGSs) were available for all isolates. Integrons were detected in the WGSs with the program IntegronFinder and the results compared with three established methods: (i) polymerase chain reaction detection of the integrase gene; (ii) BLAST searching using draft genomes; and (iii) mapping of short reads. No integrons were found in any of the Murray Collection isolates; however, integrons were present in 3% of the isolates from ECOR collection, assembled in the 1980s, and 26% of the isolates from the 2010s. Similarly, antimicrobial resistance determinants were not present in the Murray Collection isolates, whereas they were present in 19% of the ECOR Collection isolates and in 55% of the isolates obtained in during the 2010s.

## Introduction

Infections with antibiotic resistant bacteria are a leading cause of in-hospital mortality at present. Therapeutic failure in infections with Enterobacteria producing extended spectrum β-lactamases (ESBL) accounts for two thirds of hospital mortality in Europe at the time of writing, and this number was increasing [1]. Mobile genetic elements include transposons, insertion sequences and gene cassettes in integron structures all of which mobilise antibiotic resistance elements within or among DNA molecules. In consequence of this horizontal gene transfer, human pathogens are effectively enabled to acquire preexisting resistance determinants from an extensive bacterial gene pool, and thus gain effective defense mechanisms to withstand antimicrobial chemotherapy [2].

Integrons are widely present in environmental bacteria and form an important repertoire for adaption to pollution. In comparison to pristine soils, anthropogenic contamination with antimicrobials and biocides has led to enrichment of integrons with resistance markers in biocide-exposed bacterial populations [3][4]. Considering the very high levels of pollutions of soils and water in many regions, biocide contaminated soils a major concern is the generation of a large pool of integron structures with resistance determinants. Ghaly et. al. proposed, that class 1 integrons evolved through successive recombination events. This produced 3’ and 5’CS elements that are today typically found as part of larger mobile platforms like transposons and plasmids in pathogens. Thus, class 1 integrons with 3’ and 5’CS elements represent a potential source of resistance determinants for Enterobacteria [5][6][7]. These ideas help to explain why class 1 integrons increased in clinical collections of *E*. *coli*, whereas their occurrence in a historical collection of *E*. *coli* was low [8][9][10]. The present study aims to extend the understanding of the content of class 1 integrons of three *E*. *coli* isolate collections covering 100 years. Two historical isolate collections are publicly available, the Murray collection (1910s to 1940s) and the *E*. *coli* reference collection (ECOR collection; mainly 1980s) and these are complemented by a recently established collection from 2016 [11][12][13]. All collections are whole genome sequenced using Illumina technique and are available through ENA/SRA/DDBJ databases.

**Fig 1:**
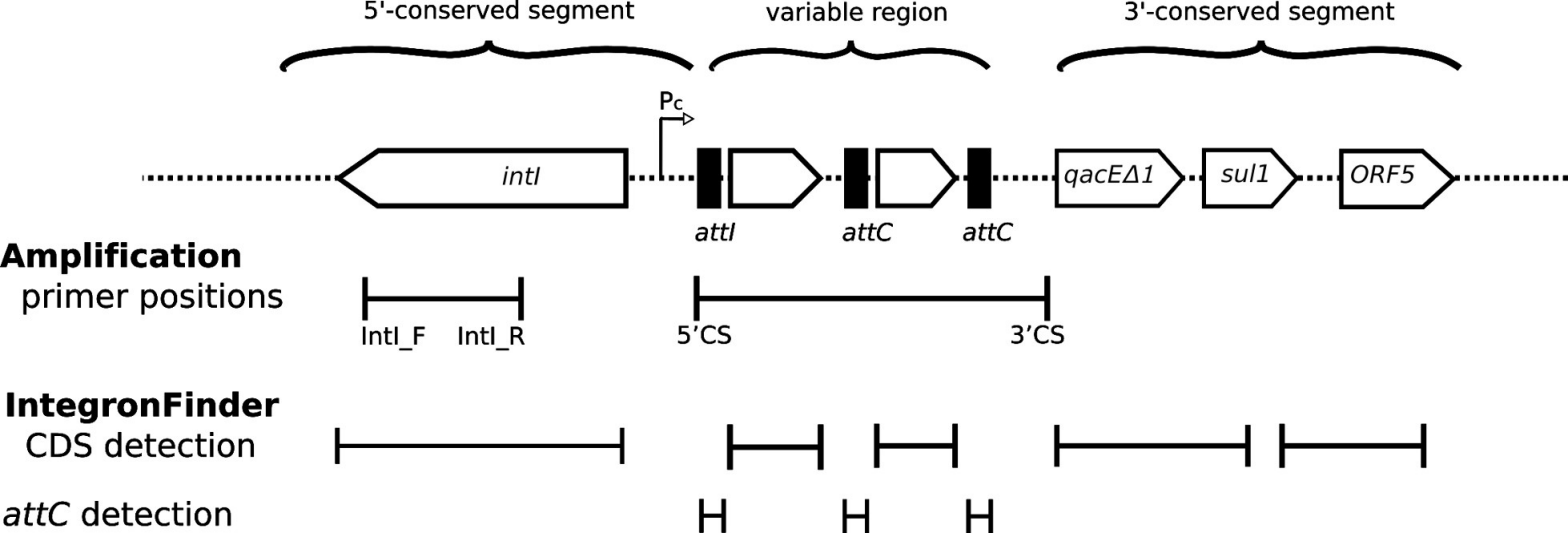
Schematic diagram of an integron, showing the location of genetic elements and detection targets for PCR analysis and IntegronFinder. The central block represents a generalized integron structure, showing the relative locations of the integrase gene *intI*, promoters, *attC* sites, and flanking the gene cassettes. The variable 3’-conserved and 5’-conserved region are indicated (top). Targets for commonly used amplification primer pairs are shown (second from bottom) along with the target structures identified by IntegronFinder (bottom).

The standalone program IntegronFinder published in 2016 predicts integrons and integron related structures in bacterial genomes [14]. The general structure of integrons is characterized by the presence of an integron-integrase gene (*intI*), a recombination site (*attI*) and one or two promoters that mediate the expression of the gene cassettes. Gene cassettes are separated by recombination sites (*attC*). IntegronFinder uses a covariance model for searching for *attC* sites and integron-integrases, and aims to complete each hit for the other parts of the integron structure. While it is known that IntegronFinder works well for whole genomes, it is less clear how it performs on draft genomes from short read sequence technologies that produce known difficulties with mobile genetic elements [14]. Conventional analysis of integrons in isolates utilizes polymerase chain reaction and commonly targets integrase gene *intI* and for the gene cassette 3’CS and 5’CS. (Fig 1) However, it is known that commonly used primer pairs can miss integron structures [15].

The present study investigated the occurrence of class 1 integrons and antimicrobial resistance determinants in three human isolate collections of *E*. *coli* covering a 100-year period. In order to do so, this study needed to evaluate conventional polymerase chain reactions on isolates as well as corresponding analyses on both short reads and draft genomes to describe the integron content in bacterial isolates.

## Material and methods

### Bacterial isolates

Whole genome sequences of three previously established *E*. *coli* isolate collections were employed in the study: (i) All *E*. *coli* isolates from the Murray collection (n = 60; available under the ENA/SRA/DDBJ databases project reference PRJEB3255) [12]; (ii) all human-derived isolates from the *E*. *coli* reference collection from the 1980s (ECOR, n = 37) [11] and (iii) a collection of *E*. *coli* from human urine samples isolated in 2016 (n = 88; ENA/SRA/DDBJ databases project reference PRJEB17631) [13]. The isolates are geographically diverse: the Murray isolates and the isolates of the ECOR collection were globally retrieved, and the isolates from the recently established collection originated from Sweden, Germany and Spain (Spain – España, herein after referred to as SGE) (S1 Table).

### General sequence analysis

Paired-end reads of the isolates from all the datasets were assembled using VelvetOptimiser software (v2.2.4) with kmer lengths from 21 to 99 using default optimization functions, and contigs were deposited at a project BIGsDB database for *E*. *coli* [16]. Species confirmation and purity of all samples was performed using the rMLST tool available at http://pubmlst.org/rmlst. This tool searches for exact matches to sequences defined in the rMLST allele library, which are derived from more than 7,000 bacterial species. Allelic matches are cross-referenced with a large curated set of bacterial isolates to determine the most likely species present in the DNA sample. Draft genomes were annotated with the annotation software prokka (v1.12-beta) [17] using the complete genome of *E*. *coli* M1655 (NCBI U00096.3) as reference for trusted proteins. Illumina short reads were mapped to databases for resistance genes (ARG-ANNOT, V2 (December 2015)) [18] for antibiotic resistance genes using srst2 (v0.2.0) [19].

#### Replicon types

Mobile genetic elements are known to be potentially affected by storage, sub-cultivation and the lack of previous, but often unknown selective pressure *in-vivo*. Of concern is the potential loss of plasmids and other mobile elements, which might confound the findings of the present work. The plasmid content can be determined in whole genome sequences by searching for their replicon sites, and is used in this study as a measure for potential effects resulting from differing storage conditions on the results of the integron analysis [20]. For all isolates, the plasmid content was analyzed through mapping of the Illumina reads to the PlasmidFinder database using srst2 with default settings, and the distribution of the number of replication types was compared among the datasets [19][20].

#### Phylogeny

All isolates were assigned to the main *E*. *coli* phylogenetic groups on the basis of their clustering in a neighbor-joining tree from rMLST-alleles. Using the BIGSdb software within the PubMLST.org website, multi-locus sequence typing was performed according to the seven gene Achtman MLST scheme [16][21]. A neighbor joining tree was constructed for rMLST allele nucleotide sequences of the study isolates and the ECOR reference strain collection [22][11]. Concatenated sequences for the rMLST scheme were retrieved through BIGsDB, aligned with MAFFT (v7.271) and the tree was calculated using PHYLIP (v3.695) [23][24]. Paralogous loci were excluded (BACT000060, BACT000065) resulting in 51 concatenated ribosomal loci for the rMLST. The dataset was then bootstrapped 500 times with PHYLIP SEQBOOT, followed by calculations of distance matrices with PHYLIP DNADIST and neighbor joining trees with PHYLIP NEIGHBOR and a consensus tree using PHYLIP CONSENSE.

### Analysis of integron related structures

#### Analysis of whole genome extracts

For the ECOR collection, the integron content was previously analyzed using amplification on whole genome preparations [10]. For the SGE collection, extracted whole genome DNA was available from previous whole genome sequencing; it had been stored in -20°C for about one year. Isolates from the SGE collection were analyzed for integron-integrase class I using the same amplification protocol that was previously used for the ECOR collection [9]. In brief, each PCR contained 2 μL template DNA, 1 μL of 10 μM each primer and TaqMix (Qiagen, Germany) to a final volume of 25 μL. The primers used had the following sequences: for integrase class forward IntI_F 5’-GGT CAA GGA TCT GGA TTT CG-3’ and reverse IntI_R 5’-ACA TGC GTG TAA ATC ATC GTC-3’, expected to give an amplification product with a size of 483 bp, and for amplification of the variable region 5'CS 5’GGC ATC CAA GCA GCA AG and 3'C 3’AAG CAG ACT TGA CCT GA with variable product size [9]. The reaction mixtures were processed in a Thermal Cycler 2720 (Applied Biosystems, USA) with the following conditions: One cycle of 12 min at 94°C; 30 cycles of 30 s at 94°C, 30 s at 62°C, 1 min at 72°C, respectively; one cycle of 8 min at 72°C. Some 5 μL of the PCR products were separated by gel electrophoresis on a 1% agarose gel stained with GelRed (Biotum, USA) and compared with a molecular weight marker (LifeTechnologies, USA) after photographing in ultraviolet light.

#### Analysis of reads

Illumina short reads were available for all three datasets and were used to map to the reference gene previously used for primer design for *intI1* gene (NCBI accession U49101) by using srst2 (v0.2.0) [19]. The result was interpreted as positive when the minimum coverage was over 90%, maximum divergence under 10% and a maximum number of mismatches per read of 10 (default settings).

#### Analysis of draft genomes

Integrase genes were searched for in the draft genomes from all data sets using nucleotide Basic Local Alignment Search Tool (BLAST) and IntegronFinder [25][14]. Nucleotide BLAST was performed for the *intI1* gene (NCBI accession U49101) using a word size of ten, match/mismatch scores of 1/-2, the gap cost was linear, and the filter was set for low complexity regions; results were considered positive when the identity of a hit was over 90%. Integron structures were identified using IntegronFinder with default setting and the local_max flag, as recommended by the developers [14]. Preparation of input files and parsing of the output files was accomplished with custom python scripts (available upon request). The classification of the results from IntegronFinder was done according to the developers: complete integrons containing integrase and *attC* sites, In0 elements with integrase gene without *attC* sites and CALIN (clusters of *attC* sites lacking integron-integrases). Additionally, open reading frames that were predicted by IntegronFinder, were annotated manually by similarity search of both nucleic acids and amino acids in the resistance gene database ARG-ANNOT, V3 (March 2017), NCBI (https://blast.ncbi.nlm.nih.gov/Blast.cgi), ENA/SRA/DDBJ (https://www.ebi.ac.uk/ena), and uniprot (http://www.uniprot.org).

### Graphical presentation of the results

Graphical illustrations of the results were produced using the package ggplot2 for heatmaps and alluvial diagrams as implemented in R (R Foundation for Statistical Computing, Vienna, Austria, http://www.R-project.org/, version 3.4.4, 2018).

## Results

### General properties of the datasets examined

After species verification using rMLST speciation tool, two isolates from the Murray collection were excluded: isolate M379 because the rMLST analysis resulted in two distinct alleles for all rMLST loci, indicating a mixture of two *E*. *coli* isolates; and isolate M126, which was identified as *Morganella morganii* in the rMLST speciation tool. Therefore, the Murray collection used here comprised 58 isolates.

#### Reads and draft genomes

The average coverage of the high-quality short reads from all collections was 112 (standard deviation [SD] of ± 24). Draft genomes were obtained for all isolates included with a median contig number (> 10,000 bp size) of 87 (range 41–153) for the Murray collection 93 (range 48–178) for ECOR and 42 (range 14–103) for the SGE collection. Accordingly, the median N50 value was 77,808 (range 25,932–298696) for the Murray isolates 74,087 (range 14,889–148,178) for the ECOR isolates and 209,521 (range 59,334–681,908) for the SGE isolates. The average of the total length of nucleotides assembled in the draft genome (> 0 bp) was 5,081,702 bp (SD ± 246,165 bp), and the median number of predicted unique genes was 4,802 (range 3,947–5,611). Compared to the reference strain K12, the median aligned genome fraction of each isolate was 85% (range 79–95%).

#### Replicon types

**Fig 2:**
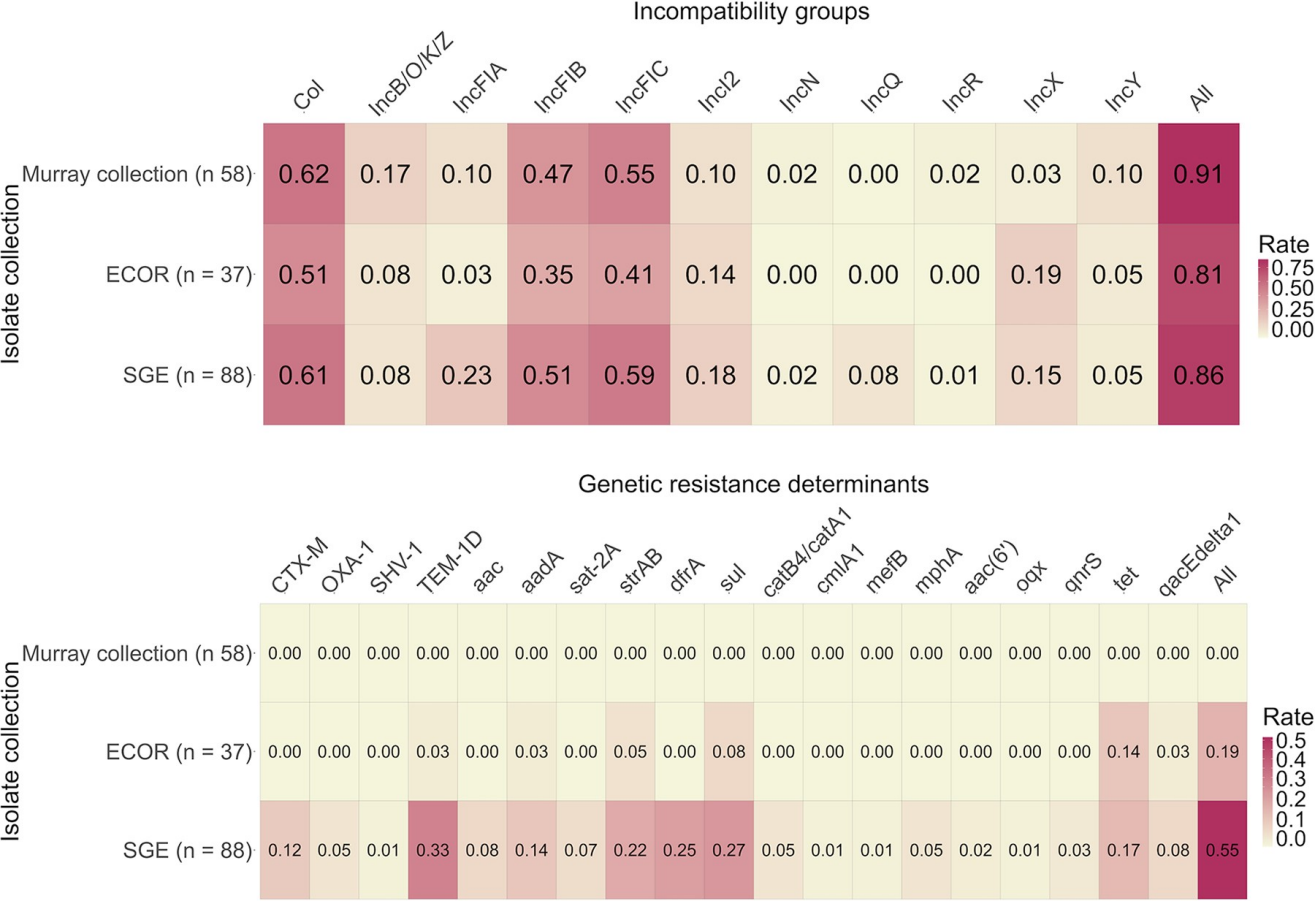
Heat maps illustrating the proportion of isolates with particular plasmid replicon types (2a) and resistance determinants (2b), by isolate collection. The proportion of isolates in which plasmid replicons for particular incompatibility groups were detected (horizontal axis) are shown by collection (panel 2a), with the last column on the right indicating the proportion of isolates with at least one plasmid detected. Panel 2b shows the presence of each resistance determinant (horizontal labels) by isolate collection, with the last column on the right giving the proportion of isolates with at least one resistance determinant in each collection. In both panels, low values appear in light colors with higher rates in dark red.

The overall occurrence of replicon types and thus incompatibility groups for plasmids, was comparable for all three datasets. At least one replicon type was found for 53/58 isolates in the Murray collection (91%), for 32/37 ECOR isolates (86%), and 76/88 isolates in the SGE collection (86%). The median count of replicon types per isolate for the Murray collection was two (range 0–8), for ECOR two (range 0–8), and for SGE collection three (range 0–9). The distribution for particular replicon types was also comparable for all three datasets (Fig 2).

#### Phylogeny

**Fig 3:**
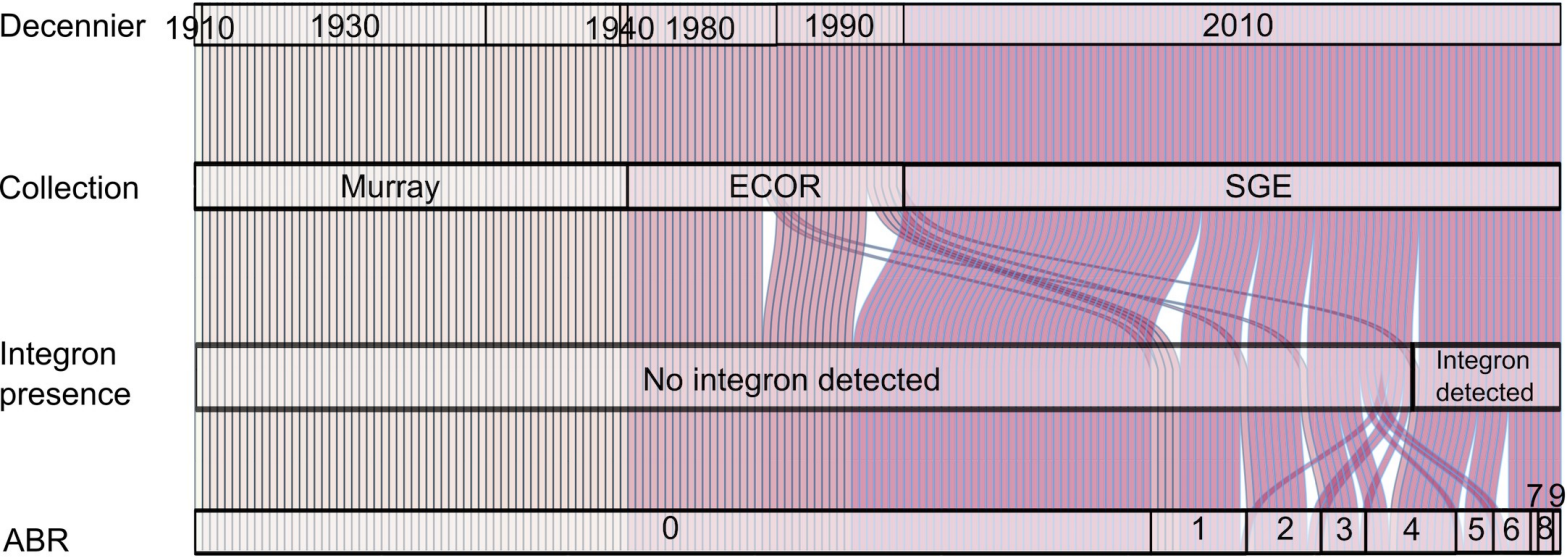
Alluvial diagram illustrating the provenance and the presence of integrons and resistance determinants in each isolate. The properties of isolates are shown in vertically from left to right with lines corresponding to: decade of isolation (top); membership of collection (second from top); presence of integron element (second from bottom), and the number of antimicrobial resistance elements found (bottom). The location of each isolate in adjacent rows is indicated with pink lines.

All *E*. *coli* phylogroups were represented among the isolates, which were dominated by and phylogroups B2 and D that are frequently associated with extra-intestinal manifestations. Within phylogroup B2, the sequence type 73 clonal complex (CC ST73) was present in all collections (n = 19), just as were CC ST1193 (n = 8) and CC ST95 (n = 7). In contrast, ST5261 (n = 6) was only found within the Murray isolates and CC ST131(n = 18) was only present in the SGE collection from 2016. The distribution of other phylogroups, namely A, C, D, F and E, was rather equal among the isolates of all collections, with the exception of ST757 (n = 5) and CC ST5308 (n = 7) that were only present in the Murray collection (Fig 3).

### Analysis of class 1 integrons

Polymerase chain reactions of class 1 integrons were performed on isolates from the SGE (n = 88) collection using the same DNA samples used for the generation of the WGS data. A total of 18 isolates gave amplification products for *intI* with a size of 483 bp. For 12 isolates, the polymerase chain reaction with primers CS’3-CS’5 resulted in amplification products of varying size (750 bp to 2000 bp), and for the remaining 76 isolates no amplicons were generated. Read mapping for *intI1* on paired-end reads of all collections resulted in reported hits for 18 isolates. BLAST search for *intI1* was performed on all draft genomes, and resulted in 16 hits, with hits in an additional two identified when using less stringent parameters (WTCHG_251, WTCHG_272). IntegronFinder was run on all 183 isolates and predicted at least one *attC* site in 180 isolates (98%) and integrase gene without *attC* sites (In0 elements) in three isolates. Complete integrons were reported in 14 isolates (8%), and *attC* sites with at least one predicted protein and lacking integron integrases (CALIN) for 135 isolates (74%). Most isolates had one (n = 34) or two (n = 47) *attC* sites, but up to nine *attC* sites were in a single isolate. The rate of detection of CALINs in each dataset was similar: 72% in Murray isolates (42/58); 68% in ECOR isolates (25/37); and 77% in the SGE isolates (68/88). All isolates but one isolate (WTCHG_282) classified as complete integrons, contained genes coding for antimicrobial resistance, and reassembled typical integron cassette contents. Isolate WTCHG_282 had an integron structure typed as complete with a partial *intI* gene (92% coverage) and a partial single IS26 element as predicted protein within its *attC* sites. Seven isolates contained CALINs with antimicrobial resistance genes typically found in integron structures, and one isolate with ribosomal genes *rpoB* and *rpoC* (WTCHG_287). In four isolates with CALIN structures the gene cassettes *aac(6’)-IId – bla*_*OXA-1*_ *– catB4* were detected, all these isolates belonged to sequence type ST131 and three had additionally *bla*_*CTX*-M-15_ coding for extended spectrum beta-lactamases. However, the majority of genes found within CALIN structures coded for proteins associated with cell structure and cell function (*rpoB/C* (n = 36); *thiI/xseB* (n = 40); *cysG/yhfL* (n = 20); ORF coding for an inositol phosphatase (n = 15); *ISEc12* (n = 31); others (n = 21)); these genes were always also present in isolates without reported CALINs.

#### Comparison of methods

Polymerase chain reaction and read mapping for class 1 integron resulted in congruent results for all 183 isolates. BLAST search gave the same result for 181 out of 183 isolates. The two isolates not detected by BLAST were integrase positive in both PCR and read map analysis: For isolate WTCHG_272 the BLAST hit covered 68% of the total length of the reference sequence and was thus not reported: the same observation was made for isolate WTCHG_282 with a coverage of only 44%. Compared to amplification and read mapping, IntegronFinder reported congruent results for 15 out of the 18 integrase positive isolates. Four additional isolates (WTCHG_211, WTCHG_236, WTCHG_248, WTCHG_258) that were not detected by the other methods had CALINS with cassette content typically found within integrons: read mapping for class 2 integron-integrase (reference L10818.1) resulted in hits for these isolates. Four isolates (WTCHG_239, WTCHG_258, WTCHG_290, WTCHG_238) had CALIN structures with the gene cassettes *aac6-IId – OXA–1 – catB4*. Of these, three were negative in the other analysis methods used. Isolate WTCHG_238 was positive for *intI* analysis with the other methods used, and had an additional In0 element on a separate node. Isolate WTCHG_216 was positive for integrase genes in all methods except for IntegronFinder, where no *attC* or In0 element was detected. Closer inspection of the contig, where the *intI* was situated revealed a class I integrase *intI* with Pc promoter and *attI* recombination site followed by *dfrA5* gene interrupted by an insertion sequence IS*26*. The contig had a coverage of 8 and was 1967 bp long. In isolate WTCHG_251, a complete integron with gene cassettes coding for *dfrA17* and *aadA5* were detected, however, the corresponding integrase gene was truncated according to read mapping and BLAST analysis, and not detectable by amplification. Taking the results from all four methods together, 24 isolates with integron related structures were found, and were thus included in the comparison of the isolate collections: 20 isolates with class 1 integrons and 4 with class 2 integrons.

### Distribution of class 1 integrons and antimicrobial resistance determinants in the isolate collections

Integron related structures were not detected in any isolate within the Murray collection, but were present in one isolate from the ECOR collection (ECOR-48, 3%), and 23 isolates from the SGE collection (26%). Antimicrobial resistance determinants were not detected in isolates from the Murray collection, whereas seven (19%) ECOR isolates and 48 (55%) isolates from SGE collection had at least one resistance determinant. In the ECOR collection, dihydropteroate synthase determinant *sul* was present in three isolates (8%); however, no isolates carried *dfrA* encoding dihydrofolate reductases. Tetracycline efflux pumps, encoded by *tet*, were found in five isolates (14%), and the beta–lactamase encoding element, *bla*_*TEM-1D*_, was found in one isolate.

**Fig 4:**
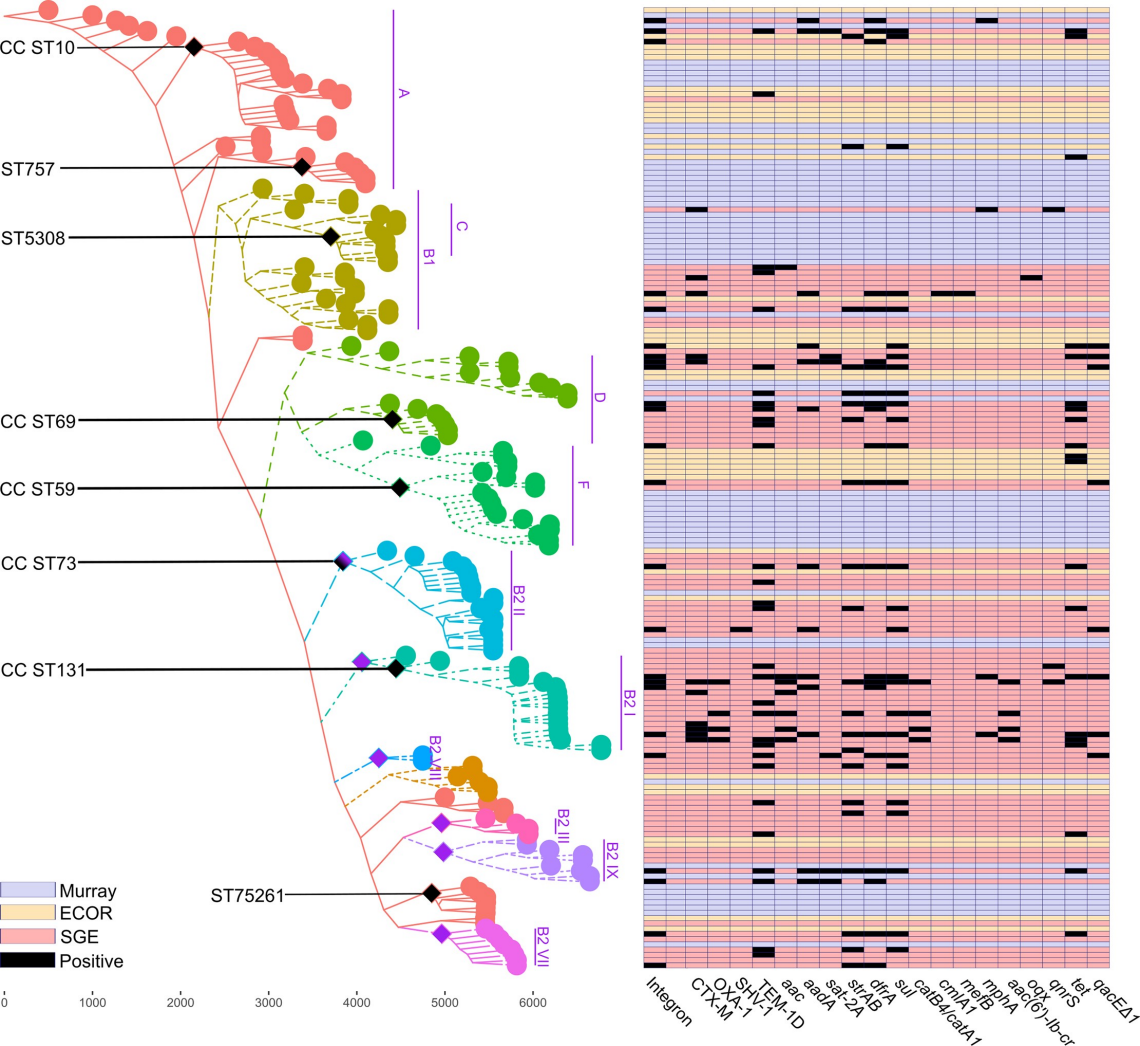
Neighbor joining phylogeny of the isolates examined (Murray, ECOR, and SGE collections) reconstructed from concatenated allele sequences of 51 ribosomal MLST loci, annotated with the presence of antimicrobial resistance determinants. In the phylogeny each tip represents one isolate, colored by lineage as labelled in purple text. Black labels indicate the corresponding MLST clonal complexes (CCs). The presence or absence of integrons and antimicrobial resistance elements are shown in the grid, with each row corresponding to the adjacent isolate in the phylogeny. Columns show the presence of each element in a given isolate in black, from integrons on the left to resistance determinants on the right, labeled along the bottom of the grid. Background colors, corresponding to the isolate collection that the isolate originated from, indicate the absence of each element, as follows: Murray collection (blue); ECOR collection (yellow); and SGE collection (red).

Within the SGE collection, folate synthesis inhibitor determinants were found in a total of 31 isolates: (i) *dfrA* encoding dihydrofolate reductases type A was present in 22 isolates; and *sul* encoding dihydropteroate synthases was found in 27 isolates. Resistance determinants for folate inhibitors *dfrA* were present in 15/24 integrons, in contrast no *sul* genes were found within integron related structures. Beta-lactamases were present in all phylogroups, extended spectrum beta-lactamases of type *bla*_*CTX*-M_ were found in eleven isolates (*bla*_*CTX*–M–15_ = 6, *bla*_*CTX*–M–9_ = 2, *bla*_*CTX*–M–1_ = 1, *bla*_*CTX*–M–27_ = 1, *bla*_*CTX*–M–55_ = 1), *bla*_*OXA-1*_ was found in four isolates, *bla*_*SHV–1*_ in one isolate and *bla*_*TEM–1D*_ in 29 isolates. The beta-lactamases *bla*_*CTX–M–15*_ and *bla*_*OXA–1*_ were more frequently present in isolates belonging to clonal complex ST131. Aminoglycoside resistance determinants were found in 19 isolates and were part of 18/24 integrons (aminoglycoside acetryltransferases *aac* (n = 7) and aminoglycoside-3''-adenylyltransferase *aadA* (n = 13)). Resistance determinants for streptothricin (*sat2A*) were found in six isolates and 21 isolates were positive for streptomycin (*strAB*); additionally, *sat2A* genes were always found as part of the variable region of integrons, but not *strAB*. Other resistance determinants that were detected, were: *catB4/catA1* (n = 4); *cmlA1* (n = 1); *mefB* (n = 1); *mphA* (n = 4); *aac(6’)-Ib-cr* (n = 4); *oqx* (n = 1); *qnrS* (n = 3); *tet* (n = 20); and *qacEΔ1* (n = 8) (Fig 4). All isolates with integron structures carried at least one antimicrobial resistance determinant; however, presence of integrons was a poor overall marker for prediction of resistance determinants (sensitivity 0.44, standard deviation ± 0.14; 0.95 confidence interval) (Figs 2, 3 and 4).

## Discussion

The present study investigated the occurrence of integron related structures in WGSs from two historical and one recent isolate collections of *E*. *coli*. The most recently *E*. *coli* collected from human disease, isolated in the 2010s had a higher occurrence of integrons with 26% of the isolates, while isolates from the ECOR collection (collected in the 1980 and 1990s) had integrons in 3% of the isolates, and there were no isolates with integrons within the Murray collection (isolated from 1910s to 1940s). Likewise, antimicrobial resistance determinants were more common in recently collected *E*. *coli*: 55% of the isolates from the 2010s had at least one resistance determinant, 19% of the ECOR isolates (1980/90s) and none from Murray (1910s – 1940s). Although at the onset of the study it was thought likely that the historical isolates would have a lower occurrence of integrons, it was striking that all isolates in the Murray collection were negative for integron-related structures.

The increase in occurrence of integrons, especially class 1 integrons is commonly discussed in the light of anthropogenic pollution of environments, including the widespread and large-scale use of antibiotics in human health care and animal food production [26][4][8][9]. While quaternary ammonium compounds can act as powerful promoters of mobilization of integrons and thus increased the occurrence of integrons in bacteria exposed to these agents, these findings are not that obvious among human clinical isolates [3]. Quaternary ammonium compounds are widely used as surface disinfectants in hospital environments and preservatives in consumer products, and they are likely exerting a selective pressure on bacterial population [27][28][26]. Despite these previous findings, none of the integron-positive isolates in this study, contained gene cassettes with functional *qac* genes [7]. On the other hand, most human infections are acquired outside the hospital environment where the selective pressure may differ. Deduction from the gene cassette content observed indicated that the predominant antibiotic resistances were those against folate inhibitors and aminoglycosides, antibiotics frequently used in animal food production [15]. Dietary intake as a source of antibiotic resistance in humans has been highlighted before, and may contribute to the persistence of the antibiotic resistance determinants despite attempts within healthcare to reduce its occurrence [29]. Class 1 integrons are known to have a low fitness cost for bacteria, because they are effectively regulated by the cellular stress levels, and thus contribute to their mobilization and spread [30][31]. However, mobilization, acquisition and stable maintenance of integrons require ongoing selective pressure, and such elements can be integrated into the genome and thus constitute a stable element in an isolate’s genome without little or no fitness cost to the bacterium [32][33].

Both antibiotics and biocides are widely used in health care, and recent interest has focused on monitoring the presence of integrons in human pathogens to complement to the local surveillance of antimicrobial resistance [34][35]. There are further benefits of screening for integrons: when integron negativity was demonstrated, the likelihood of overall antimicrobial susceptibility in an isolate was high [36], which may help reduce the use of broad-spectrum antibiotics. As of now, the detection of integron structures is not regularly employed in the characterization of routine clinical isolates since there is a lack of definitions for their applications and interpretation. Current routine methods of integron detection are polymerase chain reaction and dye-terminator sequencing, which are time- and labor intensive, with a low cost/benefit ratio. However, the recent rapid development of next generation sequencing techniques along with reductions in cost put WGS and subsequent analysis of bacterial genomes into the reach of clinical application. Automated analysis of integron structures in WGS data may therefore be an important part of routine analysis pipelines and workflows in the clinical setting.

The present study successfully used IntegronFinder on high-quality draft WGS, and simultaneously predicted new integron related structures, however, at the time of writing, the raw results of the analysis pipeline required manual curation and interpretation. Future iterations of similar tools may eliminate the need for these manual steps. An additional complicating factor for analysis is the proximity to insertion sequences that may impede the interpretation of the findings: insertions sequences can disrupt gene cassettes and thus potentially lead to loss of functionality of the determinant [37].

One potential limitation of this study was the long-term storage of the isolates in the historical collections, which could result in the loss of mobile genetic elements like transposons or plasmids, where integrons are typically located. However, both the Murray and ECOR isolate collections have previously been investigated with regard to mobile genetic elements, and no significant impact of storage has been observed [38][12][10][39]. Further, *in-silico* replicon typing performed on the collection, indicated that the content of the replicon types per isolate was comparable for all collections. Thus long-term isolate storage is unlikely to have influenced the results reported here. Variation in the predominant *E*. *coli* genotypes observed among clinical *E*. *coli* isolates over time was described previously, and is reflected in the three isolate collections examined here [40]. For example, ST757, ST5308 and ST5261 were present only in the Murray collection, while ST131, a predominant clone that arose in the 2000s was not present in the historical collections [41]. Nevertheless, there was no obvious phylogenetic bias in the genotypes present in each collection (Fig 4) which provided reassurance as to the comparability of the three isolate collections in this respect.

In conclusion, these data are consistent with an increased occurrence of integrons and antimicrobial resistance determinants among pathogenic *E*. *coli* isolates over the last one hundred years. This further highlights the central role that mobile genetic elements containing integrons play in the spread of antimicrobial resistance. Continued surveillance of integrons and their gene cassettes in a wide range of bacterial isolates is therefore essential to understand the development of antimicrobial resistance, and will provide information essential for the development of appropriate and effective countermeasures, especially in health care settings. Routine WGS analysis of bacterial isolates for the presence of integrons will therefore play a pivotal role in clinical diagnostics, and despite some limitations, has great potential for understanding and combatting the spread of these important elements.

## Supporting information

S1 Table List of the identifiers for the isolates that were included in the study. (DOCX)
