# Supplementary material for: Distribution of class 1 integrons in historic and contemporary collections of human pathogenic Escherichia coli
Source: PLoS One. 2020 Jun 2;15(6):e0233315. doi: 10.1371/journal.pone.0233315 (PMC7266292; doi:10.1371/journal.pone.0233315)
Supplement: S1 Table — (DOCX) [file pone.0233315.s001.docx]

**Supporting information**

Distribution of Class 1 Integrons in historic and contemporary collections of human pathogenic *Escherichia coli*

Susanne Sütterlin, James E. Bray, Martin C.J. Maiden, Eva Tano

**Table S1.** List of the identifiers for the isolates that were included in the study.

| **Isolate collection** | **Identifieres of the isolates** |
| --- | --- |
| Murray collection | M127, M128, M143, M163, M177, M20, M228, M256, M291, M292, M293, M297, M300, M331, M380, M389, M390, M401, M41, M427, M429, M609, M610, M611A, M611B, M612, M6133, M614, M615, M616, M617, M618, M619, M620, M631, M632, M633, M634, M647, M648A, M648B, M663, M664, M676, M677, M678, M679, M680, M682, M649, M315, M323, M265, M266, M314, M245, M233, M184 |
| ECOR collection | ECOR-01, ECOR-02, ECOR-04, ECOR-05, ECOR-06, ECOR-08, ECOR-09, ECOR-10, ECOR-11, ECOR-12, ECOR-13, ECOR-14, ECOR-15, ECOR-24, ECOR-26, ECOR-28, ECOR-35, ECOR-36, ECOR-38, ECOR-39, ECOR-40, ECOR-41, ECOR-42, ECOR-43, ECOR-48/D, ECOR-49, ECOR-50, ECOR-51, ECOR-53, ECOR-55, ECOR-56, ECOR-59, ECOR-60, ECOR-61, ECOR-62, ECOR-63, ECOR-64 |
